# Nano-Infrared Detection and Identification of Bacteria at the Single-Cell Level

**DOI:** 10.1021/acs.analchem.5c01677

**Published:** 2025-04-21

**Authors:** Axell Rodriguez, Yana Purvinsh, Junjie Zhang, Artem S. Rogovskyy, Dmitry Kurouski

**Affiliations:** † Department of Biochemistry and Biophysics, Texas A&M University, College Station, Texas 77843, United States; ‡ Department of Pathobiology and Diagnostic Investigation, Michigan State University, East Lansing, Michigan 48824, United States

## Abstract

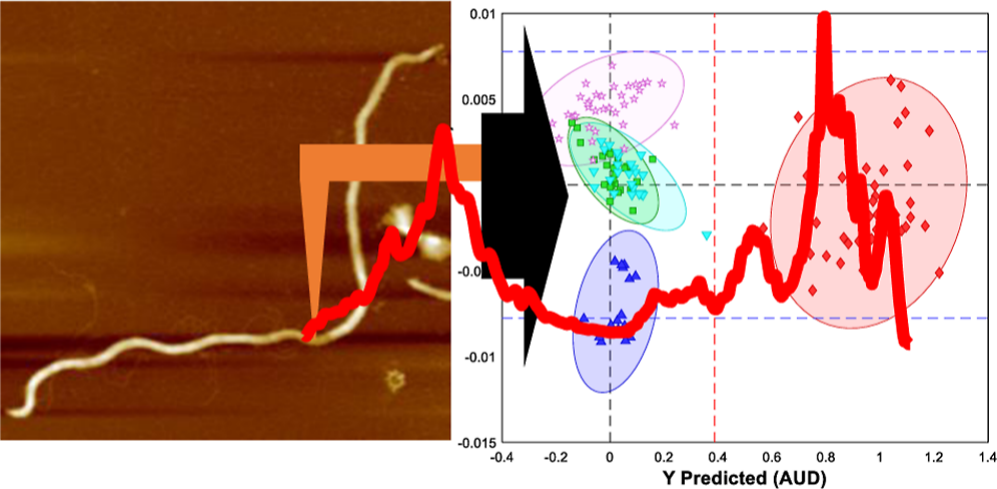

Every year, bacterial infections are responsible for over 7 million deaths globally. Timely detection and identification of these pathogens enable timely administration of antimicrobial agents, which can save thousands of lives. Most of the currently known approaches that can address these needs are time- and labor consuming. In this study, we examine the potential of innovative nano-infrared spectroscopy, also known as atomic force microscopy infrared (AFM-IR) spectroscopy, and machine learning in the identification of different bacteria. We demonstrate that a single bacteria cell is sufficient to identify *Borreliella burgdorferi*, *Escherichia coli*, *Mycobacterium smegmatis,* and two strains of *Acinetobacter baumannii* with 100% accuracy. The identification is based on the vibrational bands that originate from the components of the cell wall as well as the interior biomolecules of the bacterial cell. These results indicate that nano-IR spectroscopy can be used for the nondestructive, confirmatory, and label-free identification of pathogenic microorganisms at the single-cell level.

## Introduction

Bacterial infections put an enormous burden on global healthcare simultaneously taking over 7 million lives annually.^1^ Treatment of bacterial infections is directly linked to pathogen diagnostics. Bacterial cultures are one of the most broadly used methods to identify the specific type of bacteria causing an infection.^2^ These assays can be performed on blood, cerebrospinal fluid, stool, throat, sputum, and urine samples, while pathogen identification is based on morphology and metabolic traits of micro-organisms.^3^ Due to the required time to culture bacteria, which is the key issue in most acute bacterial infections such as sepsis, other techniques are broadly utilized. Polymerase chain reaction (PCR) is one of the molecular techniques that enables highly accurate bacterial identification.^4^ This approach requires primers against the expected pathogens and typically has difficulty in distinguishing closely related bacterial species.^4^ Furthermore, PCR is highly sensitive to contamination with organic solvents which can cause both false positive and false negative results.^5^ Some of these issues can be overcome by matrix-assisted laser desorption/ionization time-of-flight mass spectrometry (MALDI-TOF MS).^6^ This technique is based on bacterial identification based on the unique proteins present in the microorganisms.^7^ However, MALDI-TOF MS requires highly expensive instruments that are often unacceptable to most clinics and diagnostics laboratories.^8^

These limitations of traditional and molecular techniques catalyzed a search for highly accurate, less expensive, and laborious approaches that can be used for on-site pathogen identification.^9−11^ Popp and Dionne group demonstrated that bacterial traps and spontaneous Raman spectroscopy could be used for highly accurate identification of bacterial pathogens present in the blood and other body fluids.^12,13^ Similar accuracy has been demonstrated for Fourier transform near-infrared (FT-NIR)^14,15^ spectroscopy by Rodriguez-Saona and co-workers.^16^ Goodacre group showed that FT-NIR and optical photothermal infrared (O-PTIR) spectroscopy could be used to monitor the metabolism of *Escherichia coli* at the single-cell level.^17,18^ Wood group demonstrated that FT-NIR could be used to probe the chemistry of bacterial growth at the intraphase level.^19^ The same group also demonstrated that the nanoscale analogue of FT-NIR known as atomic force microscopy infrared (AFM-IR) spectroscopy could be used to monitor dynamical changes occurring in the cell wall during division of *Staphylococcus aureus* and *E. coli*.^20^ Expanding upon this, we investigate the accuracy of AFM-IR in the identification of *Borreliella burgdorferi*, *E. coli*, *Mycobacterium smegmatis,* and two strains of *Acinetobacter baumannii* at the single-cell level.

*B. burgdorferi* is a bacterial spirochete that causes a highly debilitating disease in humans known as Lyme borreliosis or Lyme disease (LD). LD is the most prevalent tick-borne disease in the Northern Hemisphere with an estimated incidence rate of approximately 500,000 cases per year in the United States alone.^21,22^ Successful treatment of LD patients with antimicrobial agents largely depends on the accurate and timely diagnosis at the early stage of this disease.^23^ Unfortunately, to date, three serological LD diagnostic approaches approved in the United States have significant drawbacks,^24−28^ accentuating the urgent need for more accurate and robust diagnostic methods.

*E. coli* is perhaps the most common pathogen often found on agricultural products as well as in water supplies. *E. coli* infection causes diarrhea, nausea, and vomiting. Global burden associated with *Mycobacterium tuberculosis* exceeds 10 million. In the US alone, 22 billion is needed annually for prevention, diagnosis, and treatment of tuberculosis caused by this pathogen.^29−31^ Due to high pathogenicity of this species of *Mycobacterium*, we utilized substantially less pathogenic form of *M. tuberculosis* and *M. smegmatis*. *A. baumannii* is a deadly pathogen that exhibits high levels of antibiotic resistance due to the sugar capsule that surrounds bacterial cells.^32−34^ Although *A. baumannii* is primarily associated with hospital-acquired infections, there are reported cases of pathogen infections in combat troops returning from conflict zones and other instances.^35^

## Results and Discussion

**Figure 1:**
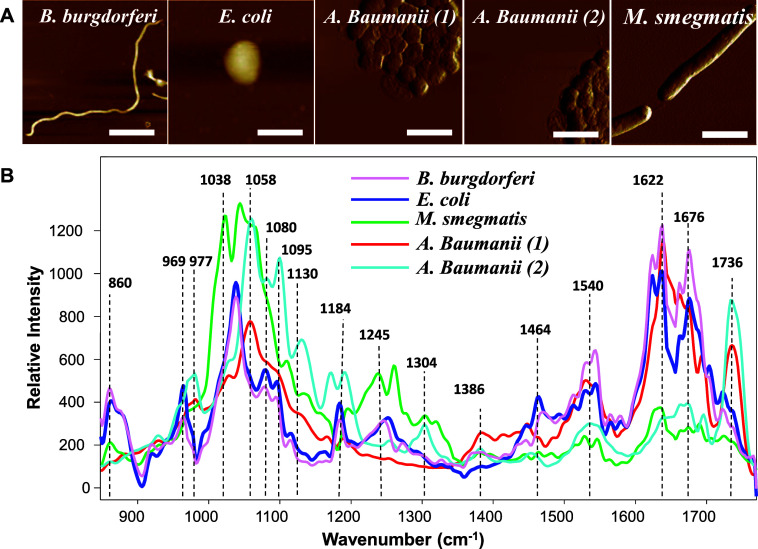
Nanoscale imaging and analysis of bacterial cells. (A) AFM images of *B. burgdorferi*, *E. coli*, *M. smegmatis,* and two strains of *A. baumannii* onto gold-coated silicon wafers. Scale bars are 5 μM (*B. burgdorferi*), 0.78 μM (*E. coli*), 3.11 μM (*A. baumannii*), and 1.71 μM (*M. smegmatis*). (B) Averaged AFM-IR spectra acquired from the bacteria.

In the current study, we deposited *B. burgdorferi*, *E. coli*, *M. smegmatis,* and two strains of *A. baumannii* onto gold-coated silicon wafers and analyzed them using AFM-IR. Microscopic imaging revealed the presence of bacterial cells with characteristic to these pathogens’ morphologies, Figure 1A. Specifically, long worm-like spirochetes were observed in the sample of *B. burgdorferi*, while almost round coccus-like bacteria were observed in the samples of *E. coli* and *A. baumannii*. Finally, elongated bacterial cells were detected upon analysis of gold-coated surfaces exposed to *M. smegmatis*.

After AFM imaging, a metalized scanning probe was placed at the bacterial cells that were localized on the analyzed surfaces. Next, the pulsed tunable IR light was used to illuminate the sample surface and induce thermal expansions in the bacterial cells that were recorded by the scanning probe and converted into IR spectra, Figure 1B.^36−42^ In the acquired spectra, we observed the vibrational bands that can be assigned to amide III (1245 cm^−1^) and I (1622−1676 cm^−1^) of proteins,^43−45^ lipids (1038−1058 and 1736 cm^−1^),^46,47^ and nucleic acids (969 and 1080 cm^−1^),^48^Table 1. We also observed a set of vibrational bands that originate from CH, CH_2_, and CH_3_ vibrations (1184, 1386, and 1464 cm^−1^) that can be assigned to any class of biomolecules, Table 1. Based on these results, we can conclude that AFM-IR detects molecules present in both cell walls and the cytosol. Specifically, we observed vibrational signatures of polyhydroxybutyrate, a biological polymer utilized by bacteria as the energy source.^49^ We also found that vibrational peaks present in the 900−1100 cm^−1^ window in the spectra acquired from *A. baumannii* could be assigned to C–O–C and C–O–H vibrations of the polysaccharide capsule^50^ that surrounds these highly antibiotic-resistant pathogens.

**Table 1:** Vibrational Bands Observed in the AFM-IR Spectra Acquired from Bacteria and Their Assignments

vibrational mode	assignment
851−860	nucleic acids [C3′-endo/anti (A-form helix)]^51^
969	nucleic acids and sugars^50,52^
1038−1058	nucleic acids and sugars (C–O–C and C–O–H vibrations)^50,52^
1080	nucleic acids (symmetric stretching P–O–C)^51^
1184	proteins (C–O stretching mode of C–OH groups)^51^
1245	nucleic acids (P–O stretching)^52^ and proteins (amide III)^53^
1386−1464	CH_2_ scissoring^54^ and C–H vibration^54^
1530−1546	proteins (amide II)^55^
1622−1690	proteins [C=O stretching (amide I)]^55^
1736	C=O, lipids^56^ and polyhydroxybutyrate^49^

**Figure 2:**
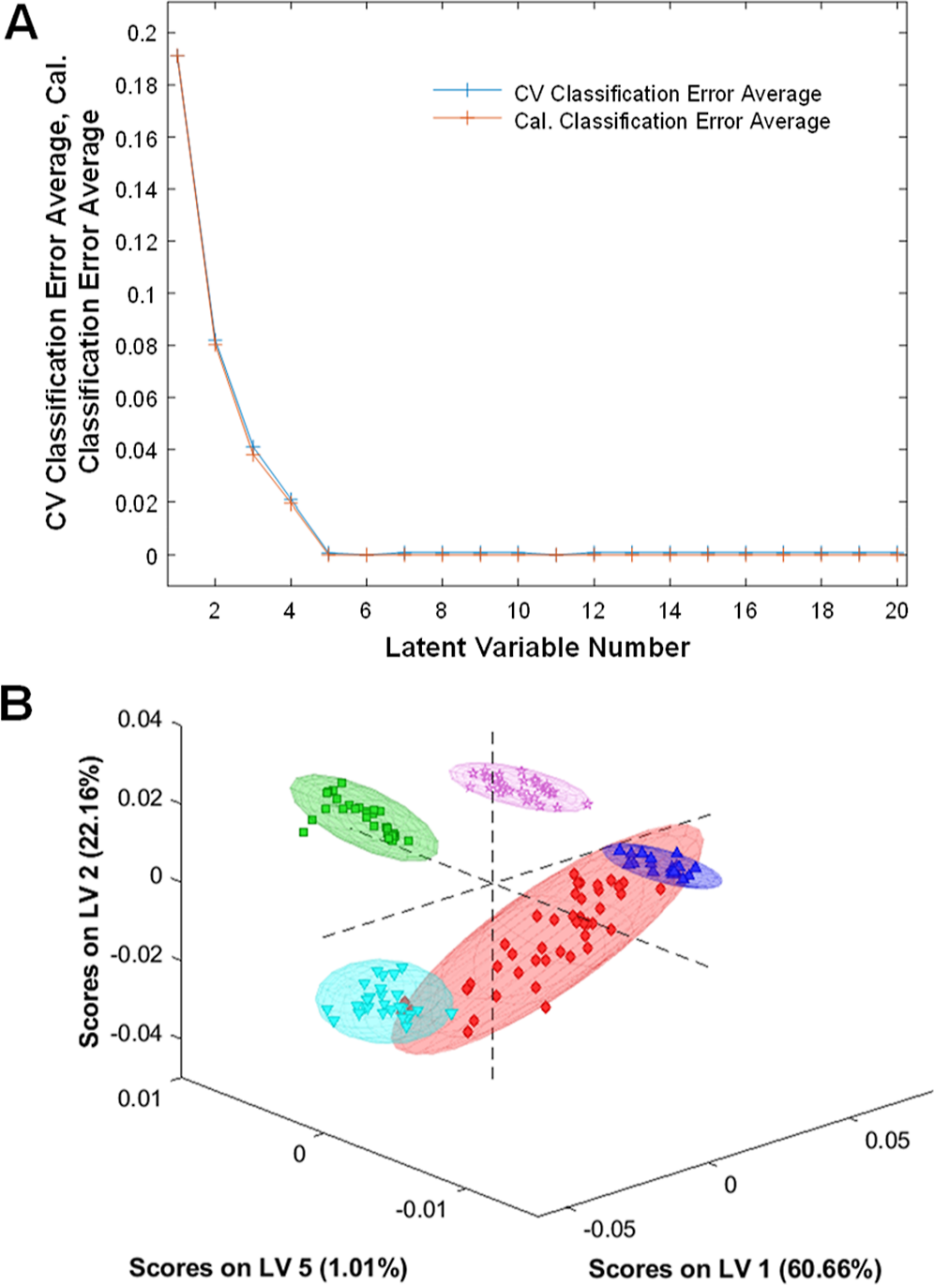
(A) Latent variable number vs CV and calculated (Cal) classification error average. (B) 3D LVs plot of acquired spectra of *B. burgdorferi* (pink), *E. coli* (blue), *M. smegmatis* (green), and two strains of *A. baumannii* (strain 1, red and strain 2, light blue); 95% confidence intervals are shown with colored ellipses.

The question to ask is whether these spectroscopic signatures can be used to identify bacterial species. To answer this question, we employed chemometrics. Specifically, the partial least-squares discriminant analysis (PLS-DA) model was built based on the 20−44 spectra acquired from each bacterial species. Our results show that PLS-DA enabled 100% accurate identification of bacterial species (Table 2 and Figure 2).

**Table 2:** Accuracy of Classification by PLS-DA for Each Class of the Acquired AFM-IR Spectra

predicted as	accuracy, %	A. baumannii (1)	M. smegmatis	E. coli	A. baumannii (2)	B. burgdorferi
A. baumannii (1)	100	44	0	0	0	0
M. smegmatis	100	0	30	0	0	0
E. coli	100	0	0	20	0	0
A. baumannii (2)	100	0	0	0	27	0
B. burgdorferi	100	0	0	0	0	35

It is important to emphasize that essentially any substrate can be used for such AFM-IR-based identification of bacteria. In our previous study, we demonstrated that gold coating on silica only enhances the IR signal by 10 times, while other substates such as calcium fluoride, zinc selenide, and sapphire can be used for the identification of bacteria. Thus, AFM-IR provides a substrate-general approach to bacterial diagnostics.

## Conclusions

Our findings demonstrate that differences in the chemical structure and composition of bacterial cell walls and their cytosol can be probed by using AFM-IR at the single cell level. The acquired spectra, in turn, can be used for the highly accurate identification of bacterial pathogens and their strains. Thus, AFM-IR can be used for a label-free, nondestructive, and confirmatory identification of bacteria.

## Experimental Section

### Bacterial Cultures

Wild-type *B. burgdorferi* strain 297 was cultivated in laboratory-made liquid Barbour–Stoenner–Kelly medium with 6% rabbit sera (Gemini Bio-Products, CA, USA) at 35 °C under 2.5% CO_2_. *M. smegmatis* strain mc^2^155 was purchased from ATCC (Lot no. 700084). Cells were grown at 37 °C in Middlebrook 7H9 medium (Difco) containing 10% (v/v) oleic acid, albumin, dextrose, catalase (OADC) supplement (Becton Dickinson), and 0.05% (v/v) Tween 80 until it reached a certain O.D. To induce a starved-phase state in *M. smegmatis*, exponentially growing cultures were harvested by centrifugation, washed, and resuspended in phosphate-buffered saline pH 7.4 with 0.05% w/v Tyloxapol, followed by incubation at 37 °C. *E. coli* [BL21(DE3)] was grown in LB broth under 37 °C and constant agitation. Two strains of *A. baumannii* were isolated from a bloodstream of an individual infected with the pathogen and grown in TSB media overnight at 37 °C. After the culture reached an OD of 0.7, 1 mL of the bacterial culture was centrifuged at low speed for 1 min. The pellet was resuspended in ethanol/acetic acid (3:1) and incubated at room temperature for 15 min. Next, bacterial cells were centrifuged at low speed for 1 min. Ethanol (96%) was added to the formed pellet and gently stirred for 5 min. Finally, bacterial cells were centrifuged at low speed for 1 min; the pellet was washed with PBS and used for AFM-IR studies.

### Atomic Force Microscopy Infrared Spectroscopy

Bacterial cultures were first diluted using DI water and then exposed to 70 nm gold-coated silicon wafers. After bacterial solutions were exposed on the wafer surfaces for ∼20 min, the excess of bacterial cultures was removed by wafer rinsing with DI water. Finally, the samples were dried at room temperature. AFM-IR imaging was performed using a Nano-IR3 system (Bruker, Santa Barbara, CA, USA). QCL laser was used as an IR source. Contact-mode AFM tips (ContGB-G AFM probe, NanoAndMore) were used to acquire the AFM images and AFM-IR spectra. No evidence of sample deformation or degradation was observed upon AFM imaging using contact-mode scanning probes. Prior to imaging, AFM-IR was optimized using a poly(methyl methacrylate) standard sample in the 860−1800 cm^−1^ spectral region. Totally, 20 spectra were acquired from different bacterial (10−20 in each sample) cells observed in each sample. To remove the artifact originating from the chip-to-chip transitions, spectra were preprocessed using MATLAB. Savitzky–Golay smoothing was applied to all spectra with 2 polynomial orders prior to the chemometric analysis.

Prior to running the PLS-DA model, area normalization and mean centering processing were applied to the spectra. Area normalization was applied to provide standardization across spectra, allowing for a more focused analysis of the relative intensities between the classes. Additionally, mean centering removes baseline offsets to remove any bias caused by artifacts present in the data scale, improving the model’s performance. PLS-DA models were built using PLS_toolbox (eigenvector Research Inc.) based on the acquired spectra that were uploaded in the MATLAB as CSV files. The calibration error shown in Figure 2A represented as a red curve depicts the error on the calibration set, also known as the training set, which reflects the model’s fit to the data presented. Analyzing this, we observe that the model performs well in the first LVs (1–4), suggesting the capture of relevant variability and overall improvement in the model’s ability to discriminate between classes. The plateau beginning at LV 5 up to LV 20 indicates that there is no improvement in the model’s performance. Thus, 5 LV were chosen, ensuring that the model is efficient and effective.
